# Supplementary material for: A Systematic Review of Qualitative Research Literature and a Thematic Synthesis of Older LGBTQ People’s Experiences of Quality of Life, Minority Joy, Resilience, Minority Stress, Discrimination, and Stigmatization in Japan and Sweden
Source: Int J Environ Res Public Health. 2023 Jul 3;20(13):6281. doi: 10.3390/ijerph20136281 (PMC10341585; doi:10.3390/ijerph20136281)
Supplement: Supplementary file 1 [file ijerph-20-06281-s001.zip › Qualitative_assessment_supplemantary file.pdf]

Supplement material Qualitative assessment

Authors (Year)	Overall assessment	Using which theory or philosophical approach did the authors proceed?	Does the research aim correspond with the research question?	Participants
Jönsson & Siverskog (2012)	Not the right phenomenon; High concerns. Not included.	Social construction framework	Unclear. The research question appears to have been formulated after finding the comments in the material, and not before data collection. There is no analysis of the group of participants per se; the comments on age are not distinctive to LGBTQ communities per se but occur in cisgender heterosexual settings as well.	Profiles on online dating sites. The selection of the sample was based on self-mocking comments in LGBTQ people above the age of 60. Only 15-30% of the participants employed self-mocking comments about their age.
Siverskog (2014)	High concerns; included for alignment with review's phenomenon of interest	Social gerontology, queer theory and social work with a biographical approach using life story interviews. It is unclear whether this has been applied regarding methodology.	Yes. This study is a substudy of a larger study; aim of larger study not described.	Trans participants taken from the larger study (which included LGBTQ people). Recruitment procedures are unclear. Concerns with recruitment (e.g., replicability). Same participants as in Siverskog (2015).
Siverskog (2015)	High concerns; included for alignment with review's phenomenon of interest	Critical gerontology, queer theory, feminist theory	Yes. This study is a substudy of a larger study; aim of larger study not described.	Trans participants taken from the larger study (which included LGBTQ people). Recruitment procedures are unclear. Concerns with recruitment (e.g., replicability). Same

				participants as in Siverskog (2014).
Löf & Olaison (2018)	Insignificant concerns. Included.	Fraser’s status model of recognition.	Yes.	Recruitment clearly described and suitable. No concerns.
Siverskog & Bromseth (2019)	High concerns; included for alignment with review’s phenomenon of interest	Queer theory and critical gerontology, ethnographic study, Ahmed’s concept of orientation, Valentine’s timespaces	Yes.	Participant data picked from two other studies; unknown sampling.

Supplement material Qualitative assessment continued

Authors (Year)	Data collection	Analysis	Researcher (s)	Relevance	Coherence
Jönsson & Siverskog (2012)	Two LGBTQ-aligned web-based internet forums: participants' profile texts were assessed and included if deemed self-mocking	Quantitative content analysis. Authors have not described the analytical process. No description of a reflexive approach. No description of how interpretations were validated.	The authors have not described their background or their relation to, or pre-understanding of, the topic.	Because there is no analysis about the comments from an LGBTQ point of view, and associating LGBTQ views in relation to age, the study is not relevant to our aims.	Only part of the data was used, as a majority of the participants did not make comments on their ages. No description whether there were contradicting findings. Analysis from a social constructive framework, but unclear how this was associated with the data.
Siverskog (2014)	Concerns, as an interview guide was not included.	Steps in thematic analysis not described. No description of a reflexive approach. No description of how interpretations were validated.	The authors have not described their background or their relation to, or pre-understanding of, the topic.	Study is relevant for the review.	Unclear whether all data were used in the analysis (because of re-use of participants' accounts in other studies). No description of whether there were contradictive data.
Siverskog 2015	Some concerns, no interview guide (replicability)	Data analysis clearly described. No description of a reflexive approach. No description of how interpretations were validated.	Author has described her background and pre-understanding. Re-use of participants or participant data not discussed.	Study is relevant for the review.	Unclear whether all data were used in the analysis (because of re-use of participants' accounts in other studies)
Löf & Olaison (2018)	Data collection clearly described.	Steps in thematic analysis clearly described. Reflexive interpretations. No description of how interpretations were validated.	Authors have described their backgrounds and pre-understanding.	Study is relevant for the review.	Most of the data were used in the analysis; contradictive data accounted for; data support results.

Siverskog & Bromseth (2019)	Data collection is unclear. No description of procedures. Authors selected data from the two other studies to be included in this one; unclear criteria for selection.	Thematic analysis was applied on selected quotes; procedure for analysis not supported by the reference provided. No description of reflexive approach. No description of how interpretations were validated.	Authors have not described their pre-understanding. Re-use of participant data not discussed.	Study is relevant for the review.	The form of data collection and analysis leads to high concerns for the trustworthiness of the study.
