# Supplementary material for: A Systematic Review of Qualitative Research Literature and a Thematic Synthesis of Older LGBTQ People’s Experiences of Quality of Life, Minority Joy, Resilience, Minority Stress, Discrimination, and Stigmatization in Japan and Sweden
Source: Int J Environ Res Public Health. 2023 Jul 3;20(13):6281. doi: 10.3390/ijerph20136281 (PMC10341585; doi:10.3390/ijerph20136281)
Supplement: Supplementary file 1 [file ijerph-20-06281-s001.zip › Review protocol_Swedish_Japanese_literature_resubmission.pdf]

Review title

A systematic review of qualitative research literature and a thematic synthesis of older LGBTQ people's experiences of quality of life, minority joy, resilience, minority stress, discrimination and stigmatization in Japan and Sweden

Japanese title:

日本とスウェーデンにおける LGBTQ 高齢者が持つ生活の質、マイノリティジョイ、レジリエンス、マイノリティストレス、差別、スティグマについての経験に関する文献のシステマティックレビューによる質的調査

Swedish title:

En systematisk översikt av kvalitativ forskningslitteratur och en tematisk syntes över äldre hbtq-personers erfarenheter och upplevelser av livskvalitet, minoritetsglädje, motståndskraft (resiliens), minoritetsstress, diskriminering och stigmatisering i Japan och Sverige

Start date

The first Japanese searches were conducted in September 2021 and the first Swedish searches were conducted in January 2022.

Anticipated completion date

January 2023.

Stage of review at time of this submission

	Started	Completed
Preliminary searches	X	X
Piloting of the study selection process	X	X
Formal screening of search results against eligibility criteria	X	X
Data extraction	X	X
Risk of bias/ quality assessment	X	X
Data analysis	X	X

Named contact

Anna Bratt, PhD anna.bratt@lnu.se

Linnaeus University, Department of Psychology, 351 95 Växjö, Sweden

Phone: +46470 76 78 20

Review members´ and organizational affiliations

Anna Bratt, PhD, Department of Psychology, Linnaeus University, Sweden

Richard Huntley, Department of Psychology, Linnaeus University, Sweden

Matilda Wurm, PhD, School of Law, Psychology and Social Work, Örebro University, Sweden

Ann-Christine Petersson Hjelm, PhD, Associate professor, Department of Business Studies; Commercial law, Uppsala University, Sweden

Tsukasa Muraya, PhD, Faculty of Design, Kyushu University, Japan

Yoshihisa Hirakawa, PhD, professor, Public Health and Health Systems, Nagoya University, Japan

Funding

MIRAI 2.0 – Joint seed funding of Japan-Sweden collaborative projects

Conflict of interest

No financial or personal conflicts of interest.

Review questions

What is the existing Swedish and Japanese research of older lgbtq people, aged 60 or older, regarding quality of life, minority joy, resilience, minority stress, and discrimination/stigmatization?

Inclusion criterias:

1). Peer reviewed research articles from either Sweden or Japan.

2). Research of older lgbtq people 60 years or older with first hand experiences from the participants.

16. Searches

Swedish searches

1. AB conducted preliminary searches with the guidance of a librarian at Linnaeus University and collected keywords from relevant articles written by experts in the field of older lgbtq adults, and in combination with database-specific search terms (thesaurus). Only **peer-reviewed** articles were included in the selected databases.

2. Databases relevant to the intersection of psychology, sociology and medicine/psychiatry which are the main areas of our interest since they coincide in the topic of the review: ASSIA, Cinahl, Medline, Psych INFO, and Social Abstracts. The searches were conducted separately, one database at a time, in order to use both keywords and specific thesaurus-words, since the latter vary in the different databases. In addition Swedish research literature was searched in the Swedish databases LIBRIS, DIVA and SwePub. LIBRIS is the Swedish libraries database, and DIVA where dissertations, research publications and student essays from most Swedish universities and colleges can be found. SwePub is a database for research publications from about 40 Swedish Universities.

Japanese searches

Start date of the searches: 7th of September, 2021.

1. TM conducted preliminary searches the Database of Kyushu University Library and collected keywords from relevant articles written by experts in the field of older lgbtq adults, and in combination with database-specific search terms (thesaurus).

Not only peer-reviewed articles were included in the selected databases.

2. Japanese Articles, relevant to

CiNii Articles

CiNii Articles, NII Scholarly and Academic Information Navigator, provides academic paper information, concentrating mainly on Japanese language academic papers.

Japan Medical Articles Society

Contains periodicals in the fields related to medicine, dentistry and pharmacy published by universities, academic societies, research institutes and hospitals in Japan.

PubMed

It is the largest life sciences literature database and is mainly based on MEDLINE literature provided by the National Library of Medicine in the United States.

Scopus

One of the world's largest databases of abstracts and citations, covering over 25,000 titles in science, technology, medicine, social sciences, and the humanities.

17. URL to the International search strategy

ASSIA 48 results

ti,ab,su(Lesbian* OR Gay OR bisexual* OR Transgender* OR Transgenderism OR Queer* OR lntersex* OR Intersexuality OR LGBTQ* OR LGBT OR LGBTQI OR LGB OR homosexu* OR "gender identity" OR "sex role identity" OR "gender minorit*" OR "sexual minorit*" OR "gender nonconformi*" OR "gender non-conformi*" OR transsexual* OR Transexual* OR "gender nonbinary" OR "gender non-binary" OR TGNC OR "Third gender" OR agender) AND (ti,ab("old* age" OR ageing OR aging OR older OR elder* OR senior*) OR (MESH.EXACT("Aging") OR (MESH.EXACT("Aged") OR MESH.EXACT("Aged, 80 & over")) OR MESH.EXACT("Aged, 80 & over")) OR (MAINSUBJECT.EXACT("Ageing") OR MAINSUBJECT.EXACT("Elderly people") OR MAINSUBJECT.EXACT("Older people") OR MAINSUBJECT.EXACT("Older women") OR MAINSUBJECT.EXACT("Older men") OR MAINSUBJECT.EXACT("Very old"))) AND ti,ab,su(discriminat* OR stigma* OR Stigmatization OR ageis* OR stereotyp* OR Prejudice*) AND (ti,ab,su((Qualitative) NEAR/1 (method* OR stud* OR research OR analysis OR data)) OR ti,ab,su(("semi-structured" OR semistructured OR "in-depth" OR indepth OR "face-to-face" OR "face to face") NEAR/1 (interview*)) OR ti,ab,su("focus group*" OR "interviews as topic" OR narration OR "personal narratives as topic" OR "Grounded Theory" OR Phenomenolog* OR "Thematic Analysis" OR "Narrative Analysis" OR Hermeneutic*)) AND (la.exact("ENG") AND PEER(yes))

Cinahl 189 results

TI (Lesbian* OR Gay OR bisexual* OR Transgender* OR Transgenderism OR Queer* OR lntersex* OR Intersexuality OR LGBTQ* OR LGBT OR LGBTQI OR LGB OR homosexu* OR "gender identity" OR "sex role identity" OR "gender minorit*" OR "sexual minorit*" OR "gender nonconformi*" OR "gender non-conformi*" OR transsexual* OR Transexual* OR "gender nonbinary" OR "gender non-binary" OR TGNC OR "Third gender" OR agender) OR AB (Lesbian* OR Gay OR bisexual* OR Transgender* OR Transgenderism OR Queer* OR lntersex* OR Intersexuality OR LGBTQ* OR LGBT OR LGBTQI OR LGB OR homosexu* OR "gender identity" OR "sex role identity" OR "gender minorit*" OR "sexual minorit*" OR "gender nonconformi*" OR "gender non-

conformi*" OR transsexual* OR Transexual* OR "gender nonbinary" OR "gender non-binary" OR TGNC OR "Third gender" OR agender) OR SU (Lesbian* OR Gay OR bisexual* OR Transgender* OR Transgenderism OR Queer* OR lntersex* OR Intersexuality OR LGBTQ* OR LGBT OR LGBTQI OR LGB OR homosexu* OR "gender identity" OR "sex role identity" OR "gender minorit*" OR "sexual minorit*" OR "gender nonconformi*" OR "gender non-conformi*" OR transsexual* OR Transexual* OR "gender nonbinary" OR "gender non-binary" OR TGNC OR "Third gender" OR agender) se bilaga, inte fullständigt

Medline 248 results

ti,ab,su(Lesbian* OR Gay OR bisexual* OR Transgender* OR Transgenderism OR Queer* OR lntersex* OR Intersexuality OR LGBTQ* OR LGBT OR LGBTQI OR LGB OR homosexu* OR "gender identity" OR "sex role identity" OR "gender minorit*" OR "sexual minorit*" OR "gender nonconformi*" OR "gender non-conformi*" OR transsexual* OR Transexual* OR "gender nonbinary" OR "gender non-binary" OR TGNC OR "Third gender" OR agender) AND (ti,ab("old* age" OR ageing OR aging OR older OR elder* OR senior*) OR (MESH.EXACT("Aging") OR MESH.EXACT("Aged") OR MESH.EXACT("Aged, 80 and over"))) AND ti,ab,su(discriminat* OR stigma* OR Stigmatization OR ageis* OR stereotyp* OR Prejudice*) AND (ti,ab,su((Qualitative) NEAR/1 (method* OR stud* OR research OR analysis OR data)) OR ti,ab,su(("semi-structured" OR semistructured OR "in-depth" OR indepth OR "face-to-face" OR "face to face") NEAR/1 (interview*)) OR ti,ab,su("focus group*" OR "interviews as topic" OR narration OR "personal narratives as topic" OR "Grounded Theory" OR Phenomenolog* OR "Thematic Analysis" OR "Narrative Analysis" OR Hermeneutic*)) AND la.exact("ENG")

PsychINFO 420 results

ti,ab,su(Lesbian* OR Gay OR bisexual* OR Transgender* OR Transgenderism OR Queer* OR lntersex* OR Intersexuality OR LGBTQ* OR LGBT OR LGBTQI OR LGB OR homosexu* OR "gender identity" OR "sex role identity" OR "gender minorit*" OR "sexual minorit*" OR "gender nonconformi*" OR "gender non-conformi*" OR transsexual* OR Transexual* OR "gender nonbinary" OR "gender non-binary" OR TGNC OR "Third gender" OR agender) AND (ti,ab("old* age" OR ageing OR aging OR older OR elder* OR senior*) OR (MESH.EXACT("Aging") OR MESH.EXACT("Aged") OR MESH.EXACT("Aged, 80 and over")) OR (age.exact("Aged (65 Yrs & Older)") OR age.exact("Very Old (85 Yrs & Older)")) OR (MAINSUBJECT.EXACT("Aging") OR MAINSUBJECT.EXACT("Aging (Attitudes Toward)") OR MAINSUBJECT.EXACT("Older Adulthood"))) AND ti,ab,su(discriminat* OR stigma* OR Stigmatization OR ageis* OR stereotyp* OR Prejudice*) AND (ti,ab,su((Qualitative) NEAR/1 (method* OR stud* OR research OR analysis OR data)) OR ti,ab,su(("semi-structured" OR semistructured OR "in-depth" OR indepth OR "face-to-face" OR "face to face") NEAR/1 (interview*)) OR ti,ab,su("focus group*" OR "interviews as topic" OR narration OR "personal narratives as topic" OR "Grounded Theory" OR Phenomenolog* OR "Thematic Analysis" OR "Narrative Analysis" OR Hermeneutic*)) AND (la.exact("ENG") AND PEER(yes))

Social Abstracts 50 results

ti,ab,su(Lesbian* OR Gay OR bisexual* OR Transgender* OR Transgenderism OR Queer* OR lntersex* OR Intersexuality OR LGBTQ* OR LGBT OR LGBTQI OR LGB OR homosexu* OR "gender identity" OR "sex role identity" OR "gender minorit*" OR "sexual minorit*" OR "gender nonconformi*" OR "gender non-conformi*" OR transsexual* OR Transexual* OR "gender nonbinary" OR "gender non-binary" OR TGNC OR "Third gender" OR agender) AND (ti,ab("old* age" OR ageing OR aging OR older OR elder* OR senior*) OR (MESH.EXACT("Aging") OR MESH.EXACT("Aged") OR MESH.EXACT("Aged, 80 & over")) OR (MAINSUBJECT.EXACT("Aging") OR MAINSUBJECT.EXACT("Elderly") OR MAINSUBJECT.EXACT("Older people"))) AND ti,ab,su(discriminat* OR stigma* OR Stigmatization OR ageis* OR stereotyp* OR Prejudice*) AND (ti,ab,su((Qualitative) NEAR/1

(method* OR stud* OR research OR analysis OR data)) OR ti,ab,su(("semi-structured" OR semistructured OR "in-depth" OR indepth OR "face-to-face" OR "face to face") NEAR/1 (interview*)) OR ti,ab,su("focus group*" OR "interviews as topic" OR narration OR "personal narratives as topic" OR "Grounded Theory" OR Phenomenolog* OR "Thematic Analysis" OR "Narrative Analysis" OR Hermeneutic*)) AND (la.exact("ENG") AND PEER(yes))

Total international searches: n= 955

Swedish searches:

LIBRIS 41 results of which all were book chapters and not peer reviewed articles

(LHBTQ OR HBT OR HBTQ* OR HBTQI OR GLBT* OR homsexue* OR lesbisk* OR bisexuel* OR trans OR transperson* OR queer* OR intersex* OR transsexualism OR könsidentitet* OR könsuttryck*) (äldre* OR ålder* OR åldrande* OR senior* OR gamling*)

SwePub 26 results of which 3 were peer reviewed articles (Siverskog 2014, 2015 and 2019)

(LHBTQ OR HBT OR HBTQ* OR HBTQI OR GLBT* OR homsexue* OR lesbisk* OR bisexuel* OR trans OR transperson* OR queer* OR intersex* OR transsexualism OR könsidentitet* OR könsuttryck*) (äldre* OR ålder* OR åldrande* OR senior* OR gamling*)

DIVA 71 results of which 15 were peer reviewed articles

HBTQ OR homsexue* OR lesbisk* OR bisexuel* OR trans OR transperson* OR queer* OR intersex* OR transsexualism

Swedish searches: n=138

Japanese Search

CiNii Articles

Result 5

（LGBT OR LGBTQ OR 性的マイノリティ OR 性的少数者 OR セクシュアル・マイノリティ） AND （ケア OR 介護） AND 高齢

Result 22

（LGBT OR LGBTQ OR 性的マイノリティ OR 性的少数者 OR セクシュアル・マイノリティ） AND 高齢

Japan Medical Articles Society

Result 15

（LGBT or LGBTQ or 性的マイノリティ or 性的少数者 or セクシュアル・マイノリティ） （ケア or 介護） 高齢

Result 56

（LGBT or LGBTQ or 性的マイノリティ or 性的少数者 or セクシュアル・マイノリティ） 高齢

PubMed

Result 11

(LGBT OR LGBTQ OR "sexual minority" OR "sexual minorities") AND ("older adults" OR Elderly OR "aged people" OR senior) AND (Japan OR Japanese)

Scopus

Result 4

(LGBT OR LGBTQ OR "sexual minority" OR "sexual minorities") AND ("older adults" OR Elderly OR "aged people" OR senior) AND (Japan OR Japanese)

In total 113 Japanese articles

In total 1206 articles

Duplicates

433 articles

Screening for eligibility: 773 articles

Excluded:

Not Swedish or Japanese research= 545 articles

Not peer reviewed: 120 Swedish and 98 Japanese papers= n= 218

Not the right age group= 1 Swedish (Kottorp 2016) + 1 Japanese (Kitajima & Sugisawa 2018) = n=2

Not older LGBTQ adults, included healthcare professionals (Smolle & Espvall 2021) and 7 Japanese papers = n=8

Selected: 5 Swedish articles 0 Japanese

Included in the synthesis= 4 papers, one paper was excluded because it was not about the lgbtq experiences only, rather age-related issues.
